# Nanomedicine Reformulation of Chloroquine and Hydroxychloroquine

**DOI:** 10.3390/molecules26010175

**Published:** 2020-12-31

**Authors:** David M. Stevens, Rachael M. Crist, Stephan T. Stern

**Affiliations:** Nanotechnology Characterization Laboratory, Cancer Research Technology Program, Leidos Biomedical Research, Inc., Frederick National Laboratory for Cancer Research sponsored by the National Cancer Institute, Frederick, MD 21702, USA; dstevens5654@gmail.com (D.M.S.); cristr@mail.nih.gov (R.M.C.)

**Keywords:** chloroquine, hydroxychloroquine, nanomedicine, nanoformulation

## Abstract

The chloroquine family of antimalarials has a long history of use, spanning many decades. Despite this extensive clinical experience, novel applications, including use in autoimmune disorders, infectious disease, and cancer, have only recently been identified. While short term use of chloroquine or hydroxychloroquine is safe at traditional therapeutic doses in patients without predisposing conditions, administration of higher doses and for longer durations are associated with toxicity, including retinotoxicity. Additional liabilities of these medications include pharmacokinetic profiles that require extended dosing to achieve therapeutic tissue concentrations. To improve chloroquine therapy, researchers have turned toward nanomedicine reformulation of chloroquine and hydroxychloroquine to increase exposure of target tissues relative to off-target tissues, thereby improving the therapeutic index. This review highlights these reformulation efforts to date, identifying issues in experimental designs leading to ambiguity regarding the nanoformulation improvements and lack of thorough pharmacokinetics and safety evaluation. Gaps in our current understanding of these formulations, as well as recommendations for future formulation efforts, are presented.

## 1. Introduction

Chloroquine (CQ) and hydroxychloroquine (HCQ) have been used for decades in the prevention and treatment of malaria and in the treatment of some autoimmune diseases such as lupus erythematosus and rheumatoid arthritis due to their immunomodulatory properties [1,2,3]. Despite being considered old drugs, CQ and HCQ have generated new interest due to their anticancer activity both in preclinical and clinical studies [4,5]. Researchers have shown these drugs act through a variety of antineoplastic mechanisms such as autophagy disruption, tumor vessel normalization, immunomodulation, and inhibition of metastasis, acting both directly on the tumor parenchyma and tumor microenvironment [6,7]. Chloroquines have been shown effective either as monotherapies or as adjunct therapies, sensitizing cancer cells to existing cytostatic agents as well as targeted therapies [7]. For example, HCQ has been shown to synergize with MEK pathway inhibitors for effective treatment of RAS-driven cancers, and CQ has been shown to inhibit melanoma growth through modifying tumor-associated macrophage (TAM) from the M2 immunosuppressive/pro-tumor phenotype to M1 immunostimulatory/antitumor phenotype [8,9].

CQ and HCQ have also recently received worldwide attention due to their potential use in treating coronavirus disease 2019 (COVID-19) caused by severe acute respiratory syndrome coronavirus 2 (SARS-CoV-2) infection. Previous studies showed in vitro efficacy of these drugs against Middle East respiratory syndrome coronavirus (MERS-CoV) and severe acute respiratory coronavirus (SARS-CoV), and a recent study demonstrated CQ could effectively inhibit viral infection of SARS-CoV-2 in vitro [10,11,12]. As a result, scientists suggested their assessment in patients, leading to emergency use authorization for HCQ and the initiation of several clinical trials. However, interest in these drugs sharply declined following a retrospective analysis claiming COVID-19 patients were more likely to die of irregular heart rhythms when taking CQ or HCQ, resulting in revocation of the FDA’s emergency use authorization [13,14]. This report was later retracted due to data validity concerns; however, many clinical trials had already been terminated. It should also be noted that recent data have questioned the original in vitro findings supporting inhibition of viral replication by CQ, demonstrating that the CQ-sensitive viral activation mechanism in the Vero cell line utilized was not relevant to human lung cells [15]. For these reasons, the use of these drugs for the prevention or treatment of COVID-19 remains extremely controversial.

CQ and HCQ are both basic amphiphiles that concentrate in the lysosome and inhibit lysosomal function as their primary mechanism of action [16]. While CQ and HCQ also have similar toxicity profiles and are equipotent, chloroquine is much more toxic (2-fold) [16]. Although short-term administration of either drug is generally well-tolerated, except in patients predisposed to arrhythmia, chronic dosing and high-dose regimens can cause severe side effects such as irreversible retinal toxicity [17,18,19]. CQ and HCQ have similar pharmacokinetic (PK) properties, including high volume of distribution and prolonged plasma half-lives between 40 and 50 days, which requires weeks of dosing to achieve steady-state therapeutic concentrations [20]. Reformulation of CQ and HCQ to improve their PK and safety profile may support the use of these drugs for applications such as cancer and infectious diseases.

**Figure 1:**
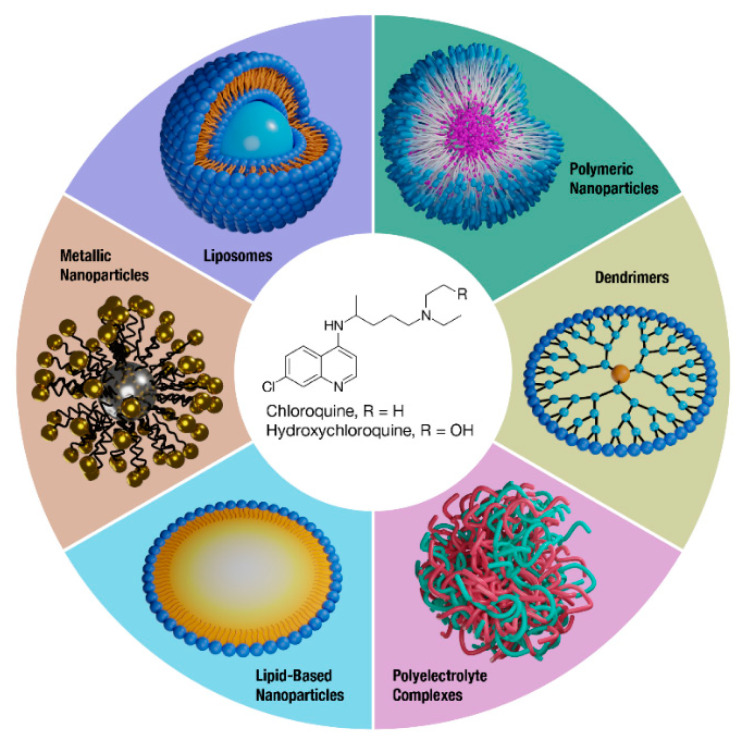
Nanomedicine formulations of CQ and HCQ. A variety of nanotechnology platforms are being explored in the reformulation efforts of improving the overall safety and efficacy of CQ and HCQ.

Nanoparticle drug delivery is one promising strategy to overcome drug liabilities such as poor PK and toxicity while improving site-specific drug delivery. Nanomedicines can provide a variety of benefits, such as improving the solubility of hydrophobic drugs, protecting drugs from degradation, and altering tissue distribution through passive or active targeting mechanisms [21]. Indeed, various nanomedicines have been developed and clinically approved that enhance the safety and/or efficacy of drugs and legacy formulations [22]. Overall, CQ and HCQ therapy may benefit from reformulation, and this review will discuss the efforts to formulate these drugs through nanomedicine approaches (Figure 1).

## 2. Liposomes

Liposomes are spherical vesicles consisting of one or more phospholipid bilayers and are capable of loading drugs within their aqueous core or lipid bilayer. Liposomes are generally very stable with long circulatory half-lives, and changes to their surface chemistry, such as hydrophilic coating (e.g., polyethylene glycol; PEG) or targeting moieties (e.g., antibodies), can result in decreased uptake by the mononuclear phagocytic system (MPS) and site-specific delivery, respectively [23,24]. Liposomal formulations of CQ, with and without erythrocyte-specific antibody targeting fragments, were first developed during the 1980s and provided better suppression of parasitemia compared to unformulated CQ in malaria parasite *P. berghei*-infected animals (Table 1) [25,26,27,28,29,30]. Despite these early successes, liposomal CQ did not progress toward clinical applications, and only a few liposomal CQ formulations have been published since. For example, Fotoran et al. developed micron-sized, multilamellar liposomes for loading CQ through interlayer hydrogen bonding [31]. In comparison to unformulated CQ, this formulation only provided a significant reduction in parasitemia for two of the thirteen-day efficacy study, suggesting only a modest improvement in therapy.

It is worth noting that these studies utilized non-PEGylated liposomes, which are known to be rapidly cleared by resident macrophages in MPS organs such as the liver and spleen [32]. Although this is unfavorable for many applications, since it lowers drug exposure to non-MPS tissues, some researchers have utilized non-PEGylated liposomes as a strategy to increase drug exposure to macrophages and improve treatment of macrophage-based infections. For example, in a *C. neoformans* murine model, liposomal CQ in combination with fluconazole provided better antifungal prophylaxis and treatment compared to free drug controls due to enhanced liposomal drug uptake by macrophages [33,34]. Most modern liposomal formulations contain a PEG surface coating that reduces macrophage clearance and increases circulatory time, which may be desirable for malaria and cancer indications. In one recent example, a CQ formulation using PEGylated liposomes with antibody targeting to the erythrocyte surface protein glycophorin A provided robust CQ delivery to uninfected and Plasmodium-infected red blood cells, resulting in superior efficacy compared to unformulated CQ in *P. falciparum*-infected mice [35]. Overall, these studies support the use of liposomal formulations for delivering CQ to erythrocytes and macrophages for malaria and antifungal applications, but additional PK and toxicology studies would be informative to evaluate their safety profile moving forward.

Liposomes initially found clinical success as drug carriers in cancer treatment with the development of Doxil^®^ (liposomal doxorubicin), which reduced the drug’s dose-limiting cardiotoxicity and increased tumor exposure due to the enhanced permeability and retention (EPR) effect [36]. The EPR effect concept was first introduced by Matsumura and Maeda et al. in 1986; this ability of nanoparticle-based formulations to accumulate in tumor tissue is now widely recognized and was recently reviewed by Price et al. [37,38]. In particular, liposomes have become a commonly used formulation to passively target one or multiple drugs to tumors. Due to CQ’s anticancer activity, researchers have developed liposomal formulations combining CQ and other chemotherapeutics for enhanced anticancer efficacy. For example, liposomes co-loaded with CQ and paclitaxel (PTX) or doxorubicin (DXR) resulted in tumor growth suppression in A549/T-tumor-bearing mice and MCF-7/ADR-tumor-bearing zebrafish, respectively [39,40]. However, the authors did not compare to unformulated drug controls in the efficacy or drug distribution studies, and therefore, it is unclear if the liposomal formulations provided any benefits to CQ delivery, a major shortcoming of these studies.

Several HCQ-loaded liposomes have also been developed for cancer indications. For example, Wang et al. combined HCQ-loaded liposomes with TAT-Beclin 1 peptide to induce autophagy catastrophe in a 4T1 breast cancer model [41]. The combination treatment significantly reduced tumor growth compared to unformulated drug controls and HCQ liposomes alone, suggesting improved liposomal HCQ tumor exposure and supporting the strategy of inducing autophagy catastrophe in tumor cells to treat cancer. A similar strategy was used to combine HCQ-loaded liposomes with Salmonella VNP20009 antitumor peptide in a B16F10 melanoma xenograft model [42]. The liposomes increased HCQ concentrations 4-fold within the tumor compared to the free drug control 24 h following injection, with no difference in liver or spleen concentrations at this same time point. This improvement in tumor drug exposure resulted in 90% survival in comparison to 20% survival for the free drug HCQ + VNP20009 combination control group, and no survival in the HCQ, VNP20009, or HCQ-liposome only groups. These studies strongly support the use of liposome formulations to increase HCQ delivery to the tumor site and improve efficacy when combined with other anticancer drugs.

To further improve the delivery of HCQ to tumors, liposomes have been modified with various targeting ligands to enable tumor-specific drug delivery. For example, liposomes decorated with pH-sensitive RGD peptides for targeting ITGAV-ITGB3/integrin αvβ3 receptors were used for HCQ delivery to melanoma tumors [43]. Both untargeted and targeted versions of the liposomes significantly decreased drug exposure to the heart, spleen, lung, and kidney compared to unformulated drug control, while only the targeted liposome version significantly increased HCQ concentrations within the tumor 24 h post-injection. As a monotherapy, the formulation achieved a median survival of 30 days compared to 25 days from the untargeted liposome treatment group and 15 days from HCQ free drug control. However, when combined with liposomes containing DXR, the median survival improved to >60 days, and tumor growth was significantly inhibited compared to free drug controls or DXR liposomes only. This same ITGAV-ITGB3/integrin αvβ3 receptor-targeted liposome formulation was also used to co-deliver HCQ and PTX for pancreatic cancer therapy [44]. This formulation achieved significantly better tumor growth inhibition and reduction of metastatic tumor nodules in a BxPC-3-luc orthotopic tumor model compared to targeted liposomes containing either HCQ or PTX, untargeted liposomes containing both drugs, and PTX + HCQ free drug control while not affecting body weight. This ITGAV-ITGB3/integrin αvβ3 receptor-targeted liposomal formulation not only significantly changed HCQ distribution toward the tumor, but also provided excellent anticancer efficacy when combined with chemotherapeutics.

In addition to integrin αvβ3 receptors, Yin et al. also targeted neuropilin-1 receptors on melanoma cells for co-delivery of HCQ and PTX [45]. This targeted liposomal formulation significantly inhibited tumor growth and effectively inhibited metastasis in a B16F10 melanoma model compared to an untargeted liposome version and unformulated drug controls. Another liposomal formulation, also targeting the integrin αvβ3 and neuropilin-1 receptors was co-loaded with HCQ and tyrosine kinase inhibitor ZD6474 and evaluated for efficacy in a C6 glioma model [46]. Interestingly, these liposomes did not significantly change drug exposure to the heart, liver, spleen, lung, or kidney compared to untargeted liposome version or free drug controls, but they did achieve a 4.9-fold increase in drug exposure to the brain in C6 intracranial tumor-bearing mice. This improvement in drug delivery across the blood-brain barrier (BBB) resulted in significantly prolonged median survival time with the targeted, co-loaded liposomes (41 days) compared to the untargeted liposome version (35 days) and unformulated, free drug controls (28 days).

Ultrasound (US) is another method that has been investigated as a means to improve nanoparticle delivery across the BBB and is also involved in sonodynamic therapy [70,71]. This strategy was used to improve the delivery of HCQ and sonoactive chlorin e6 to glioma tumors using angiopep-2 peptide-modified liposomes that target low-density lipoprotein receptor-related protein 1 (LRP1) [47]. Combined with ultrasonic pulse, the targeted liposome containing both drugs achieved the greatest median survival time of 52 days compared to 40 days from the untargeted liposome version and 33 days from chlorin e6 + HCQ unformulated drug controls. Combining autophagy inhibitors with sonodynamic therapy through targeted drug delivery to brain tumors may offer a novel therapeutic strategy for glioma.

Drugs that are not suitable for remote loading into the liposomal aqueous core or are not sufficiently lipophilic to associate with the lipid bilayer can be conjugated to a lipid anchor to facilitate loading within the lipid bilayer [72]. Although researchers have shown HCQ can be successfully incorporated in the liposomal aqueous core with high drug loading, Liu et al. developed a liposome bilayer-loaded cholesterol-modified version of HCQ for the treatment of pulmonary fibrosis [48]. Both cholesterol-modified HCQ liposomes and core-loaded HCQ liposomes inhibited the development of bleomycin-induced pulmonary fibrosis in Sprague-Dawley rats; however, the authors did not compare to unformulated HCQ, so the benefits of using a liposomal bilayer-loaded cholesterol-modified HCQ formulation remain unclear.

## 3. Polymeric Nanoparticles

Polymer-based nanoparticles have been used to improve the solubility of hydrophobic drugs and facilitate enhanced tumor distribution through the EPR effect. Polymeric micelles, one of several different types of polymer-based nanoparticles, generally consist of amphipathic polymers that co-precipitate with drugs to form a hydrophobic core surrounded by a hydrophilic shell. These formulations have been shown to have low critical micelle concentrations (CMC) and have better stability than traditional surfactant micellar systems due to hydrophobic interactions between the drug and polymer [73]. CQ is a hydrophobic drug with a high logP of 4.72 and is predicted to be suitable for polymeric micelle formulations based on previous analysis of how drug properties influence nanomedicine compatibility [74,75]. Despite this, few examples of CQ-polymeric micelles have been reported. In one study, micelles composed of methoxy PEG-b-poly(L-lactic acid) (mPEG-PLA) were used to co-load CQ with either DXR, PTX, or cis-platin [49]. In all cases, the micellar formulations provided superior efficacy in ovarian cancer models compared to unformulated drug combinations, indicating improved tumor distribution.

In addition to micelles, polymeric nanoparticles can be formed through emulsion techniques. This approach can be used to encapsulate hydrophilic drugs and biologics within the polymer matrix and do not require amphipathic polymers. For example, Yang et al. developed a nanoparticle composed of poly(lactic-co-glycolic acid) (PLGA) for co-delivery of CQ and pDNA expressing the mSurvivin-T34A protein [50]. In this case, CQ was used for pDNA compaction through electrostatic interactions as well as for improving lysosome escape of the pDNA following cell uptake. This formulation provided better tumor growth inhibition compared to pDNA/PLGA nanoparticle without CQ in a CT26 tumor model. However, the authors did not compare to a CQ free drug control or to pDNA/PLGA + CQ administered separately, so it is unclear if the improvement in efficacy is due simply to the addition of CQ or to an improvement in CQ drug delivery.

HCQ has also been formulated with biologics to aid in lysosome escape. For example, Liu et al. developed a PLGA nanoparticle co-loaded with HCQ and ovalbumin (OVA) as a model antigen for a proof-of-concept vaccine delivery formulation [51]. This formulation provided statistically significant tumor growth inhibition in an OVA-sensitive E.G7-OVA xenograft tumor model compared to free OVA or OVA-nanoparticles alone, but the authors did not include controls for unformulated HCQ administered alone or in combination with OVA-nanoparticles. Further studies are required to determine if there is a benefit to formulating CQ or HCQ to facilitate cytosolic delivery of biologics, or if the same effects can be achieved by simply administering the drugs separately.

Similar to liposomes, polymeric nanoparticles can be coated with antibodies to enable tumor-specific drug delivery, but few have been developed for CQ or HCQ. In one example, a cd20-antibody-targeted poly(caprolactone)/PLA nanoparticle was co-loaded with HCQ and chlorambucil and evaluated for efficacy in a Burkitt lymphoma animal model [52]. The targeted nanoparticle provided 90% survival after 120 days compared to 40% survival in animals treated with the antibody alone and 0% survival in animals treated with untargeted, drug-loaded nanoparticles or free drug combination controls. Interestingly, at non-toxic doses, the untargeted version of the nanoparticle provided worse survival (0%) compared to the free drug combination control (33%), indicating an untargeted polymeric nanoparticle may unfavorably change tissue distribution of these drugs.

Although most polymers used in drug delivery are biodegradable, some non-biodegradable polymers such as acrylamide-based polymers have shown success for small molecule and oligonucleotide delivery [76,77]. One major advantage of acrylic polymers is the wide selection of functionalized monomers available to form polymers with different physicochemical properties. For example, poly(N-isopropylacrylamide-co-acrylic acid) (PNIPAM-Aac) is a negatively charged polymer that can undergo electrostatic complexation with positively charged molecules. This approach was used to co-load CQ and DXR within PNIPAM-Aac nanogels to induce autophagy catastrophe within tumor cells [53]. Despite successful drug loading, CQ release in PBS was rapid, with more than 50% in the first two hours and more than 95% over 12 h. This rapid drug release is likely too fast to benefit from any passive tumor targeting of the nanoparticle. Indeed, in an efficacy study in an MCF-7 breast cancer model, the nanoparticles containing both drugs did not achieve a statistically significant decrease in tumor weight compared to CQ-only nanogels. The authors also did not compare to a free drug DXR + CQ control to prove the benefit of nanoparticle delivery.

Overall, due to a lack of appropriate controls, there is limited data to support the utilization of polymeric nanoparticles for improving the delivery of either CQ or HCQ.

## 4. Dendrimers

Dendrimers are repetitively branched molecules generally constructed as macromolecular polymers with variable cores and terminal groups to facilitate drug encapsulation and drug delivery [78]. Properties such as size, morphology, and surface chemistry can be controlled through synthetic chemistry steps and designed for specific drug delivery needs. To improve CQ delivery to Plasmodium-infected red blood cells, Marti Coma-Cros et al. designed cationic dendrimers based on Pluronic F127 and 2,2′-bis(glycyloxymethyl)propionic acid as well as a hyperbranched dendrimer derived from 2,2′-bis(hydroxymethyl)propionic acid [54]. Although both dendrimer formulations were capable of loading CQ and demonstrated parasite growth inhibition in vitro, they provided worse survival outcomes (20%) in *P. yoelii*-infected mice compared to CQ control (80%), indicating the formulations significantly reduced the antimalarial efficacy of CQ. One possible explanation for this decrease in efficacy could be due to a reduction in systemic drug exposure. Previously, dendrimers composed of PEG and poly(lysine) with and without galactose terminal groups significantly reduced the maximum concentration (Cmax) and area under the concentration-time curve (AUC) of CQ in comparison to unformulated CQ [55]. A similar CQ-loaded PEG-poly(lysine dendrimer) with a chondroitin sulfate A coating also significantly reduced Cmax compared to free drug (13.85 and 50.23 µg/mL, respectively), but increased AUC from 74.72 to 120.58 µg*h/mL; however, in this case the differences in PK were likely due to the routes of administration, since the unformulated drug was administered intravenously and the dendrimer formulation was administered intramuscularly [56].

Alternatively, Panagiotaki et al. designed dendrimers composed of poly(ethylenimine) with triphenylphosphate terminal groups to facilitate mitochondrial delivery of DXR and CQ for improved cancer therapy [57]. Dendrimer formulations were developed for each drug and, when administered together, significantly reduced tumor volume in DU145 tumor-bearing mice. However, the efficacy was only slightly better than the DXR-only dendrimer, and the authors did not compare to a CQ-only dendrimer formulation or DXR + CQ free drug control. Therefore, it is unclear whether this formulation provided any benefit to the delivery or anticancer efficacy of CQ.

## 5. Polyelectrolyte Complexes

Polyelectrolyte complexes, also sometimes referred to as polyplexes and coacervates, are formed by mixing oppositely charged polyionic species in an aqueous medium, and various ionic polymers have been investigated extensively for their ability to complex with nucleic acids [79]. However, their use for delivering small molecule drugs has been limited, likely due to the necessity of multiple charge sites per drug molecule to allow stable complexation with the polymer.

CQ is positively charged at physiological pH due to its two ionizable amine groups, and because of this, researchers have attempted to load the drug into complexes containing ionic polymers. In one example, Urban et al. developed poly(amidoamine) polymers that formed ~10 nm complexes when mixed with CQ [59]. Drug release from the formulations in PBS was nearly identical to unformulated CQ, indicating formulation instability. Surprisingly, *P. yoelii*-infected mice treated with the polymer/CQ complexes achieved 100% survival 30-days post-infection compared to 0% survival in the unformulated CQ control group. Although the polymers alone were shown to reduce parasitemia in vitro, polymer-only controls were not included in the in vivo efficacy study. Therefore, it is unclear whether the improved survival is due to an improvement in CQ delivery or rather due to additive or synergistic effects of the drug and polymers. Furthermore, the formulations provided no statistically significant improvement in survival compared to CQ alone in *P. yoelii*-infected mice when administered orally [58].

Another CQ-polyelectrolyte complex, composed of chitosan and tripolyphosphate, was shown to reduce parasitemia to a greater extent than unformulated CQ in several efficacy studies in *P. berghei*-infected mice [60,61,62,63]. However, the authors did not use vehicle-only controls in any of the studies to rule out the possible antimalarial activity of the polymer complex itself. Overall, these studies support the use of combining ionic polymers with CQ to improve malaria treatment since there is evidence of better survival outcomes and reduced parasitemia, possibly due to additive effects between CQ and the ionic polymers, rather than improved delivery to target cells.

## 6. Non-Liposomal Lipid-Based Nanoparticles

In addition to liposomes, there are a variety of other lipid-based nanoparticles including solid lipid nanoparticles (SLN), nanoemulsions, and niosomes. These formulations are generally used for improving the solubility and delivery of hydrophobic drugs and are highly biocompatible and biodegradable due to their physiological lipid compositions.

Unlike other lipid-based carriers, SLN contain a solid lipid core and are often utilized as oral formulations to improve solubility and intestinal absorption of hydrophobic drugs [80,81]. CQ is typically administered orally and has highly variable bioavailability ranging from 52% to 102% as an oral solution and 67–114% as a tablet [82]. It has also been shown that taking CQ with food results in significantly higher Cmax and AUC, and it is recommended to avoid an upset stomach during CQ dosing [83]. Despite having high oral bioavailability, Bhalekar et al. attempted to improve CQ oral delivery and intestinal lymphatic uptake using a SLN formulation for arthritis therapy [64]. The SLN formulation achieved 2-fold increases in Cmax, time of maximum concentration (Tmax), and AUC in comparison to standard CQ suspension, reportedly due to intestinal lymphatic uptake and bypassing first-pass metabolism. Consequently, the SLN formulation achieved greater paw volume reduction compared to the standard CQ suspension in the arthritis mouse model.

In addition to loading drugs, lipid-based carriers have been shown to inhibit malarial parasitemia in erythrocytes [84]. Due to these properties, Baruah et al. developed CQ-loaded, cationic nanoemulsions to improve antimalarial efficacy [65]. The formulation suppressed parasitemia by 99.68% compared to only 76.5% by unformulated CQ in *P. berghei*-infected mice 5 days post-infection. However, the blank lipid emulsion reduced parasitemia by 35.35%, indicating the lipid emulsion alone inhibited malarial infection. Therefore, it is unclear if the efficacy from the CQ nanoemulsion is due to an improvement in drug delivery or simply additive or synergistic effects with the lipid emulsion and drug.

Niosomes are another class of drug delivery vehicle capable of loading both hydrophobic and hydrophilic drugs. Niosomes are similar to liposomes in that they also contain a bilayer and an aqueous core. Unlike liposomes, which typically utilize phospholipids, niosomes are formed from mixtures of non-ionic surfactant molecules and cholesterol. Niosomes have been used for transdermal drug delivery due to their ability to improve drug penetration through the skin and provide local and sustained drug release [85]. This strategy was used to develop a HCQ-loaded niosome formulation dispersed in a Pluronic F-127 gel for the treatment of oral lichen planus [66]. Human patients applied the niosome gel with or without the drug (placebo group) to their lesion every day for four months. Patients receiving the HCQ-containing gel observed an average lesion size reduction of 64.28% compared to only 3.94% reduction in the placebo group. On a pain score from 0 to 10, where 0 is no pain and 10 is the worst pain, patients in the gel and placebo groups reported pain scores of 4 and 3 pre-treatment and 1 and 3 post-treatment, respectively. Although these data support the benefits of this HCQ niosome gel in human patients, the authors did not compare to HCQ gel control, HCQ free drug control, or standard of care (corticosteroids). Therefore, it is unclear whether encapsulation within niosome provided any benefits to the delivery of HCQ.

## 7. Metal Nanoparticles

Metallic nanoparticles have been successfully implemented as contrast agents and many are being investigated as therapeutic agents and drug delivery vehicles [86,87]. One of their limitations for drug delivery is the requirement of functional groups on the drug that can undergo chelation with metals. For example, thiol-containing drugs can be conjugated to the surface of gold nanoparticles through Au-thiol bonding. Upon cell entry, thiol-exchange with intracellular glutathione releases the drug. Drugs without thiol groups must be chemically modified as prodrugs in order to conjugate to gold nanoparticles and allow the release of the parent drug. Ruan et al. used this strategy to modify DXR and HCQ as ester prodrugs containing terminal thiol groups to enable coupling to gold nanoparticles and evaluated these nanoparticles for antiglioma efficacy [67]. The nanoparticles containing both drugs resulted in a 56-day median survival in C6 glioma-bearing mice compared to 44 days from nanoparticles containing only DXR; however, the results were not statistically significant. The nanoparticles containing only HCQ resulted in a 38-day median survival compared to 30 days from the free HCQ treatment group, though a better control would have been the modified version of HCQ since this is the molecule that is released from the gold nanoparticle. The authors described in vitro DXR release in PBS at acidic pH, but they did not investigate HCQ release, and drug release in plasma would be a better predictor of nanoparticle stability in vivo since plasma contains both glutathione and esterase enzymes. Therefore, the stability of the HCQ prodrug and its chelation with the nanoparticle surface are unclear.

HCQ has also been used to enhance sonodynamic therapy of metallic nanoparticles through autophagy disruption. For example, Feng et al. designed HCQ-loaded hollow mesoporous titanium dioxide nanoparticles that are coated with a cancer cell membrane to allow homologous targeting to the tumor [68]. HCQ release in PBS from coated nanoparticles was much slower than that of uncoated nanoparticles, but the release became equivalent to the uncoated particles when exposed to US irradiation, suggesting a US responsive drug release mechanism. In MCF-7 tumor-bearing mice, the cancer cell membrane coated nanoparticles extended the systemic half-life of HCQ to 12.3 ± 1.7 h, which was higher than that of uncoated nanoparticles (8.7 ± 1.3 h) and free HCQ (3.4 ± 0.4 h). However, it is unclear if the authors measured the total drug fraction in the blood or the released (pharmacologically active) fraction. The PK of nanomedicines is very complex since total drug concentration in the plasma and blood, as well as tissues, is comprised of encapsulated and unencapsulated drug fractions, and both fractions can contribute to drug efficacy and toxicity [88]. Nevertheless, the cancer cell membrane coated nanoparticles containing HCQ combined with tumor US irradiation significantly reduced tumor growth compared to empty nanoparticles + US and free HCQ controls, supporting the strategy of combining US with autophagy disruption. However, the degree to which the nanoparticle improved HCQ exposure of the tumor site remains unknown, and treatment of HCQ + nanoparticle + US may have been just as effective.

## 8. Conclusions and Perspectives

There is new interest in repurposing CQ and HCQ for novel applications such as cancers, as well as improving therapy for their traditional indications such as infectious and inflammatory diseases. Nanomedicines have been evaluated for their ability to improve the safety and efficacy of chloroquines. There are a variety of nanoparticle types, with each having their own advantages and disadvantages, and it is important to understand the liabilities and physicochemical properties of the drug being formulated in order to select the most appropriate platform. In the case of CQ and HCQ, off-target toxicities can be reduced, and efficacy enhanced using a combination of site-specific drug delivery and controlled release; the balance between delivery and release kinetics being a crucial factor in improving therapeutic index [89]. In order to achieve this, researchers have tested nearly every type of nanomedicine available, with many failing to conclusively demonstrate benefits to CQ or HCQ therapy.

Polymeric nanoparticles, which have been successful in formulating hydrophobic drugs in preclinical and clinical studies, are typically unstable formulations that release their drug immediately after injection, thereby eliminating any potential benefits of nanoparticle distribution and essentially acting as solubilizing formulations. For example, Genexol^®^ PM, a polymeric nanoparticle formulation of PTX that is approved as a cancer therapy in South Korea, has been shown to completely release its drug within 10 min after exposure to plasma [90]. Dendrimers and polyelectrolyte complexes have shown promising preclinical results for gene delivery but have been less successful in formulating small molecule drugs. Dendrimer-drug conjugates of chemotherapeutics are currently undergoing clinical trials, and this may prove to be a more useful strategy since drug release stability is controlled through the linker chemistry [91,92]. Metallic nanoparticles have been approved as contrast and therapeutic agents, but none have proven useful for improving the delivery of small molecule drugs, likely due to insufficiently stable drug-metal interactions. All of these nanoparticle types have been used to reformulate CQ and HCQ, but most have not provided sufficient evidence of improving their efficacy and safety profile. In many cases, appropriate controls were missing, and it was unclear if the efficacy of CQ and HCQ was due to an improvement in drug delivery or if the same results could be achieved using the unformulated drugs. Therefore, additional PK and efficacy studies with appropriate controls are needed to support the use of these nanomedicine formulations for CQ or HCQ delivery. Further, toxicity studies are also rarely performed on these formulations and are necessary for evaluation of improvements to the therapeutic index overall.

On the other hand, liposomal formulations appeared to provide a clear benefit to the delivery of CQ and HCQ in various malaria and tumor models, respectively. With its ionizable amine groups, CQ can be actively loaded into the aqueous liposomal core, and erythrocyte-specific targeting ligands on the surface of the liposomes improve drug uptake within red blood cells, a target for malaria. Since lipids have been shown to inhibit Plasmodium infection, combining CQ with lipid-based carriers may provide not only better drug delivery to uninfected and infected red blood cells, but also synergistic efficacy. Liposomes also make a good choice for improving the delivery of these drugs to tumors. With their ~100 nm size and good stability, liposomes are able to accumulate within the tumor microenvironment via the EPR effect and deliver their therapeutic cargo [93]. With the help of targeting peptides on their surface, liposomes were able to co-deliver HCQ and other chemotherapeutics to significantly improve efficacy and survival outcomes and appear to be a promising strategy for cancer therapy moving forward. However, one disadvantage of liposomes is that they are generally very stable with extremely long drug release half-lives. For example, Doxil has a drug release half-life greater than 100 h, and there are currently efforts to design less stable liposomes that provide faster drug release rates at the site of interest [94,95,96].

One notable absence in the above nanotechnology formulation discussion of chloroquines is polymer prodrug systems, a major drug delivery class that has scarcely been evaluated for these drugs and may offer an ideal balance of targeting and stability. Polymer prodrugs can be designed to be biodegradable, provide site-specific targeting, and enable controlled drug release through the polymer-drug linker chemistry [97]. This strategy has proven useful for the delivery of small molecule drugs for cancer and neurological diseases, and there are several candidates in clinical trials [98]. To our knowledge, only a single example of a polymer prodrug of HCQ evaluated in vivo has been published, and it demonstrated substantially better efficacy and lower toxicity compared to unformulated HCQ in a mouse model of colitis [69].

It should be emphasized that despite the promising preclinical data for some of the formulations presented in this review, none of the formulations have made it to the clinical stage. The lack of clinical development is likely due to poor intellectual property protections and uncertain commercial promise for the formulation platforms presented, many of which rely on generic formulation strategies. However, it is expected that the recent commercial success of novel nanotechnology-based delivery platforms and renewed interest in chloroquine drugs for novel indications, such as cancer, will fuel future clinical development of chloroquine nanoformulations [9,99]. Overall, reformulation efforts of CQ and HCQ through nanomedicine approaches have shown some promising improvements in efficacy and safety, but further developments are warranted.

## Figures and Tables

**Table 1:** Nanomedicine formulations of CQ and HCQ tested in vivo.

Formulation	Drug Loading Method	Drug	Co-drug	Indication	Reference
Liposome (Anti-mouse erythrocyte F(ab’)_2_ targeted- egg PC/Chol/gangliosides)	Passive loading by thin film sonication	CQ	-	Malaria	[25]
Liposome (Mouse monoclonal antibody F_10_ targeted- egg PC/chol/gangliosides)	Passive loading by thin film sonication	CQ	-	Malaria	[26]
Liposome (Soybean PC/egg PG/Chol)	Passive loading by reverse-phase evaporation, sonication, and extrusion	CQ	-	Malaria	[28,29,30]
Liposome (Anti-mouse erythrocyte Fab’ targeted-MPC-PE, Chol/PC/PS)	Passive loading by reverse-phase evaporation, sonication, and extrusion	CQ	-	Malaria	[27]
Liposome (Multilamellar- DOPC/DPGG/amine-N-[4-(p- maleimidophenyl) butyramide)])	Passive loading by thin film sonication	CQ	-	Malaria	[31]
Liposome (Glycophorin A targeted- DOPC/DSPC/DSPE-PEG2000-Mal)	Passive loading by thin film hydration, sonication, and extrusion	CQ	-	Malaria	[35]
Liposome (Egg PC/Chol)	Passive loading by thin film hydration and sonication	CQ	Fluconazole	*C. neoformans*	[33,34]
Liposome (Soybean PC/Chol)	Thin film hydration, sonication, and active loading using a citrate pH-gradient	CQ	PTX	Lung cancer	[39]
Liposome (Soybean PC/Chol)	Thin film hydration, sonication, and extrusion, followed by active loading using a citrate pH-gradient	CQ	DXR	Breast cancer	[40]
Liposome (Soybean PC/Chol/DSPE-mPEG2000)	Thin film hydration, sonication, and active loading using a citrate pH-gradient	HCQ	Tat-Beclin1 peptide	Breast cancer	[41]
Liposome (Soybean PC/Chol/DSPE-mPEG2000)	Thin film hydration, sonication, and active loading using a citrate pH-gradient	HCQ	VNP20009	Melanoma	[42]
Liposome (ITGAV-ITGB3/integrin α_v_β_3_ receptor-targeted- soybean PC/Chol/DSPE-PEG2000-Mal)	Thin film hydration, sonication, and active loading using a citrate pH-gradient	HCQ	DXR	Melanoma	[43]
Liposome (ITGAV-ITGB3/integrin α_v_β_3_ receptor-targeted- soybean PC/Chol/DSPE-PEG2000-Mal)	Thin film hydration, sonication, and active loading using a sulfate pH-gradient	HCQ	PTX	Pancreatic cancer	[44]
Liposome (Neuropilin-1/integrin α_v_β_3_ receptor-targeted- soybean PC/Chol/DSPE-PEG2000-Mal)	Thin film hydration, sonication, and active loading using a sulfate pH-gradient	HCQ	PTX	Melanoma	[45]
Liposome (Neuropilin-1/integrin α_v_β_3_ receptor-targeted- soybean PC/Chol/DSPE-PEG2000-Mal)	Thin film hydration, sonication, and active loading using a sulfate pH-gradient	HCQ	ZD6473	Glioma	[46]
Liposome (LRP1-targeted-DSPC/DOPC/DSPE-PEG2000-Mal)	Thin film hydration, sonication, and extrusion, followed by active loading using a citrate pH-gradient	HCQ	Chlorin e6	Glioma	[47]
Liposome (Chol-HCQ/PC)	Passive loading by thin film hydration and sonication	HCQ (cholesterol modified)	-	Pulmonary fibrosis	[48]
Polymeric micelle (mPEG-PLA)	Thin film hydration	CQ	DXR, PTX, cis-platin	Ovarian cancer	[49]
Polymeric NP (PLGA)	Water-in-oil-in-water (w/o/w) double emulsion solvent evaporation method	CQ	pDNA	Colon cancer	[50]
Polymeric NP (PLGA)	Water-in-oil-in-water (w/o/w) double emulsion solvent evaporation method	HCQ	OVA	Vaccine	[51]
Polymeric NP (CD-20 antibody-targeted- PCL/PLA)	Not described	HCQ	Chlorambucil		[52]
Acrylamide nanogel	Electrostatic complexation	CQ	DXR	Breast cancer	[53]
Dendrimer (Bis-MPA/glycine)	Emulsion evaporation method	CQ	-	Malaria	[54]
Dendrimer (PEG-PLL/galactose)	Equilibrium dialysis	CQ	-	Malaria	[55]
Dendrimer (PEG-PLL/chondroitin A sulfate)	Equilibrium dialysis	CQ	-	Malaria	[56]
Dendrimer (PEI/triphenylphosphate)	Precipitation	CQ	DXR	Prostate cancer	[57]
Polyelectrolyte complex (Poly(amidoamine))	Electrostatic interaction	CQ	-	Malaria	[58,59]
Polyelectrolyte complex (Chitosan/tripolyphosphate)	Electrostatic interaction	CQ	-	Malaria	[60,61,62,63]
SLN (Compritol^®^ proprietary lipid)	Melt homogenization method	CQ	-	Arthritis	[64]
Lipid nanoemulsion	Microemulsion method	CQ	-	Malaria	[65]
Niosome gel	Emulsion evaporation method	HCQ	-	Oral lichen planus	[66]
Gold NP	Conjugated to gold np via HCQ thiol prodrug	HCQ	DXR	Glioma	[67]
Titanium dioxide NP	Inclusion complex	HCQ	-	Breast cancer	[68]
Polymer prodrug(poly(N-(2-hydroxypropyl) methacrylamide-co-methacryloylated HCQ))	Polymer ester prodrug	HCQ	-	Inflammatory bowel disease	[69]

Bis-MPA: 2,2′-bis(hydroxymethyl)propionic acid; Chol: cholesterol; DOPC: 2-dioleoyl-sn-glycero-3-phosphocholine; DPGG: 1,2-dipalmitoyl-galloylglycerol; DSPC: 1,2-distearoyl-sn-glycero-3-phosphocholine; DSPE-PEG2000: 1,2-distearoyl-sn-glycero-3-phosphoethanolamine-N-[methoxy(polyethylene glycol)-2000]; DSPE-PEG2000-Mal: 1,2-distearoyl-sn-glycero-3-phosphoethanolamine-N-[maleimide(polyethylene glycol)-2000]); MPB-PE:maleimido-4-(p-phenylbutyrate)-phosphatidylethanolamine; mPEG: methoxypoly(ethylene glycol)-b-poly(lactic acid); PC: phosphatidylcholine; PCL: polycaprolactone; PEG-PLL: poly(ethylene glycol)-block-poly(L-lysine); PEI: polyethylenimine; PG: phosphatidylglycerol; PLA: polylactic acid; PLGA: poly(lactic-co-glycolic acid); PS: phosphatidylserine.

## Data Availability

No new data were created or analyzed in this study. Data sharing is not applicable to this article.
